# Oxidative Stress Induces Mitochondrial Dysfunction in a Subset of Autism Lymphoblastoid Cell Lines in a Well-Matched Case Control Cohort

**DOI:** 10.1371/journal.pone.0085436

**Published:** 2014-01-08

**Authors:** Shannon Rose, Richard E. Frye, John Slattery, Rebecca Wynne, Marie Tippett, Oleksandra Pavliv, Stepan Melnyk, S. Jill James

**Affiliations:** Department of Pediatrics, Arkansas Children's Hospital Research Institute, Little Rock, Arkansas, United States of America; University of Tasmania, Australia

## Abstract

There is increasing recognition that mitochondrial dysfunction is associated with the autism spectrum disorders. However, little attention has been given to the etiology of mitochondrial dysfunction or how mitochondrial abnormalities might interact with other physiological disturbances associated with autism, such as oxidative stress. In the current study we used respirometry to examine reserve capacity, a measure of the mitochondrial ability to respond to physiological stress, in lymphoblastoid cell lines (LCLs) derived from children with autistic disorder (AD) as well as age and gender-matched control LCLs. We demonstrate, for the first time, that LCLs derived from children with AD have an abnormal mitochondrial reserve capacity before and after exposure to increasingly higher concentrations of 2,3-dimethoxy-1,4-napthoquinone (DMNQ), an agent that increases intracellular reactive oxygen species (ROS). Specifically, the AD LCLs exhibit a higher reserve capacity at baseline and a sharper depletion of reserve capacity when ROS exposure is increased, as compared to control LCLs. Detailed investigation indicated that reserve capacity abnormalities seen in AD LCLs were the result of higher ATP-linked respiration and maximal respiratory capacity at baseline combined with a marked increase in proton leak respiration as ROS was increased. We further demonstrate that these reserve capacity abnormalities are driven by a subgroup of eight (32%) of 25 AD LCLs. Additional investigation of this subgroup of AD LCLs with reserve capacity abnormalities revealed that it demonstrated a greater reliance on glycolysis and on uncoupling protein 2 to regulate oxidative stress at the inner mitochondria membrane. This study suggests that a significant subgroup of AD children may have alterations in mitochondrial function which could render them more vulnerable to a pro-oxidant microenvironment derived from intrinsic and extrinsic sources of ROS such as immune activation and pro-oxidant environmental toxicants. These findings are consistent with the notion that AD is caused by a combination of genetic and environmental factors.

## Introduction

The autism spectrum disorders (ASD) are a heterogeneous group of neurodevelopmental disorders defined by impairments in communication and social interactions along with restrictive and repetitive behaviors [1]. An estimated 1 out of 88 individuals in the United States are currently affected with an ASD and the incidence continues to rise [2]. Despite decades of research, we have limited knowledge of the causes of ASD or the risks associated with developing ASD [3]. Recent studies have recognized that a broad range of children with ASD have impairments in several basic physiological processes such as energy generation systems [4] and redox homeostasis [5]–[7].

Mitochondrial dysfunction has become increasingly accepted as a major physiological disturbance in ASD [8]. However, the etiology of mitochondrial dysfunction is not known. Indeed, although mitochondrial deoxyribonucleic acid (DNA) mutations are commonly found in classical mitochondrial disease (MD), such mutations are found in only 23% of ASD children diagnosed with MD [8]. This raises the possibility of acquired mitochondrial dysfunction since mitochondrial damage can result from environmental exposures implicated in ASD such as heavy metals [9]–[12], exhaust fumes [13], polychlorinated biphenyls [14] or pesticides [15], [16]. Alternatively, mitochondria can be damaged by endogenous stressors associated with ASD such as elevated proinflammatory cytokines resulting from an activated immune system [17]–[19] or other conditions associated with oxidative stress [20], [21]. The notion of an acquired mitochondrial disorder is supported by a recent twin study which concluded that the environment contributes a greater percent of the risk of developing autistic disorder (55%) as compared to genetic factors (37%) with these factors contributing about equally for the broader ASD diagnosis [3].

Oxidative stress may be a key link between mitochondrial dysfunction and ASD as reactive oxygen species (ROS) generated from pro-oxidant environmental toxicants [9]–[16] and activated immune cells [4], [8] can result in mitochondrial dysfunction [8]. Four independent case-control studies have documented oxidative stress and oxidative damage in plasma, immune cells and post-mortem brain from ASD children [5], [7], [22], [23]. Interestingly, resting peripheral blood mononuclear cells (PBMC) and activated lymphocytes and monocytes from children with ASD demonstrate a significant decrease in glutathione redox balance reflecting an intracellular deficit in glutathione-mediated antioxidant and detoxification capacity in these immune cells [24].

An underlying defect in mitochondrial function could be a pivotal deficit in ASD as mitochondrial dysfunction affects high energy demanding organs, particularly the brain and immune system, and could also account for the commonly reported systemic abnormalities associated with ASD, such as immune dysfunction. Diverse immune abnormalities including abnormal lymphocyte activation [25], [26] and monocyte proinflammatory cytokine production [27]–[29] have been reproducibly reported in ASD and found to be associated with increased severity of the core and related symptoms of ASD. Indeed, immune cells can be a suitable model for investigating the consequences of mitochondrial abnormalities when nervous tissue cannot be practically studied.

We have previously demonstrated that lymphoblastoid cell lines (LCLs) derived from children with autistic disorder (AD) produce higher levels of ROS and exhibit a significant decrease in both intracellular and mitochondrial glutathione redox capacity when compared to control LCLs [22]. Furthermore, when challenged with nitrosative stress, the AD LCLs exhibit a greater reduction in mitochondrial membrane potential compared to control LCLs [22]. This evidence suggests that glutathione-mediated redox capacity is insufficient to counter endogenous ROS production in these AD LCLs resulting in increased vulnerability to oxidative damage and mitochondrial dysfunction during pro-oxidant exposures. Mitochondria are both the main producers and main targets of ROS in most cell types; however, redundant mechanisms exist to regulate excessive mitochondrial ROS production to protect electron transport chain (ETC) complexes, which can be damaged and inactivated by ROS. Uncoupling protein 2 (UCP2) is one of the major control mechanisms for reducing high levels of ROS at the inner mitochondrial membrane. In many cell types, including lymphocytes, UCP2 is up-regulated under conditions of chronic mitochondrial oxidative stress to relieve the proton gradient across the inner mitochondrial membrane and reduce mitochondrial ROS production [30]–[32].

In this study we hypothesized that a subset of LCLs derived from patients with AD are vulnerable to ROS, such that excessive intracellular ROS results in mitochondrial dysfunction. To this end, we examined mitochondrial respiratory activity in LCLs derived from AD children and age-matched unaffected controls. Specifically we concentrate our studies on reserve capacity, a measure of ability of the mitochondria to respond to physiological stress. Importantly, a reduction in reserve capacity has been linked to aging [33], heart disease [34], and neurodegenerative disorders [35], [36]. Hill et al [37] have demonstrated that reserve capacity is important for protecting the cell from acute increases in ROS, but that once reserve capacity is exhausted, cell vulnerability is increased and viability is reduced. Thus, we hypothesized that a subgroup of AD LCLs will demonstrate abnormal reserve capacity when exposed to increasing concentrations of ROS. We further hypothesized that this subgroup of AD LCLs will be more vulnerable to ROS and will exhibit an increase in intracellular and intramitochondrial mechanisms to compensate for increased ROS. To this end we measured glycolysis as representative of intracellular compensatory mechanisms and cellular UCP2 content and function as a representation of intramitochondrial compensatory mechanisms. For the first time, we demonstrate atypical changes in mitochondrial respiration when exposed to ROS in a subgroup of AD LCLs, and that this atypical AD subgroup exhibits higher UCP2 content.

## Methods

### Lymphoblastoid Cell Lines and Culture Conditions

Twenty five LCLs derived from white males diagnosed with AD chosen from pedigrees with at least 1 affected male sibling (mean/SD age 8.5±3.4 y) were obtained from the Autism Genetic Resource Exchange (Los Angeles, CA, USA) or the National Institutes of Mental Health (Bethesda, MD, USA) center for collaborative genomic studies on mental disorders (Table 1). Thirteen age-matched control LCLs derived from healthy white male donors with no documented behavioral or neurological disorder or first-degree relative with a medical disorder that could involve abnormal mitochondrial function (mean/SD age 8.8±3.7 y) were obtained from Coriell Cell Repository (Camden, NJ, USA). Due to low availability of control LCLs from children with no documented neurological disorders, we paired a single control LCL line with 1, 2 or, in one case, 3 AD LCL lines (age-matched LCL pairs are listed in Table 1). On average, cells were studied at passage 12, with a maximum passage of 15. Genomic stability is very high at this low passage number [38], [39]. Cells were maintained in RPMI 1640 culture medium with 15% FBS and 1% penicillin/streptomycin (Invitrogen, Grand Island, NY, USA) in a humidified incubator at 37°C with 5% CO2.

**Table 1:** Lymphoblastoid cell line characteristics and matching between AD and control cell lines. List is organized by the two groups identified: AD-A and AD-N.

	Autism				Control		
Pair #	CellID	Source	Age (y)	Subgroup	CellID	Source	Age (y)
1	03C14441	NIMH	7	AD-A	GM17255	Coriell	6
2	03C16499	NIMH	11	AD-A	GM15862	Coriell	11
3	1393306	AGRE	3	AD-A	GM09659	Coriell	4
4	0939303	AGRE	11	AD-A	GM15862	Coriell	11
5	1165302	AGRE	13	AD-A	GM11626	Coriell	13
6	01C08594	NIMH	7	AD-A	GM17255	Coriell	6
7	01C08495	NIMH	4	AD-A	GM09659	Coriell	4
8	02C09713	NIMH	7	AD-A	GM11973	Coriell	7
9	02C10054	NIMH	6	AD-N	GM09380	Coriell	6
10	04C26296	NIMH	10	AD-N	GM11599	Coriell	9
11	00C04757	NIMH	10	AD-N	GM10153	Coriell	10
12	05C38988	NIMH	12	AD-N	GM16007	Coriell	12
13	03C15992	NIMH	5	AD-N	GM18054	Coriell	5
14	038804	AGRE	8	AD-N	GM11599	Coriell	9
15	1267302	AGRE	10	AD-N	GM10153	Coriell	10
16	1215301	AGRE	12	AD-N	GM16007	Coriell	12
17	008404	AGRE	13	AD-N	GM11626	Coriell	13
18	02C10618	NIMH	7	AD-N	GM09622	Coriell	7
19	02C09650	NIMH	7	AD-N	GM09622	Coriell	7
20	01C08367	NIMH	7	AD-N	GM09642	Coriell	7
21	04C27439	NIMH	7	AD-N	GM09642	Coriell	7
22	03C14349	NIMH	17	AD-N	GM17272	Coriell	17
23	04C24363	NIMH	4	AD-N	GM18054	Coriell	5
24	01C08022	NIMH	5	AD-N	GM09380	Coriell	6
25	03C17237	NIMH	10	AD-N	GM10153	Coriell	10

NIMH = National Institutes of Mental Health (Bethesda, MD, USA).

AGRE = Autism Genetic Resource Exchange (Los Angeles, CA, USA).

Coriell = Coriell Cell Repository (Camden, NJ, USA).

### Seahorse Assay

We used the state-of-the-art Seahorse Extracellular Flux (XF) 96 Analyzer (Seahorse Bioscience, Inc, North Billerica, MA, USA), to measure the oxygen consumption rate (OCR), an indicator of mitochondrial respiration, and the extracellular acidification rate (ECAR), an indicator of glycolysis, in real-time in live intact LCLs.

**Figure 1:**
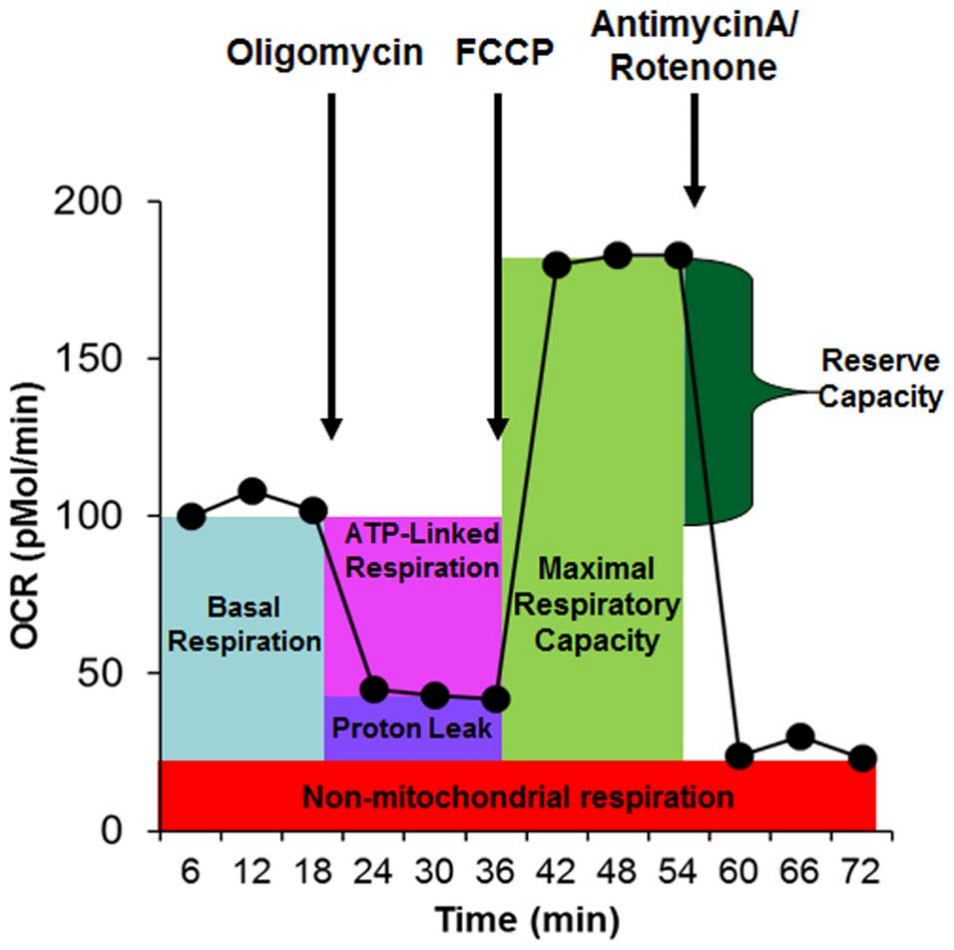
The Seahorse assay. Oxygen consumption rate (OCR) is measured before and after the addition of inhibitors to derive several parameters of mitochondrial respiration. Initially, baseline cellular OCR is measured, from which basal respiration can be derived by subtracting non-mitochondrial respiration. Next oligomycin, a complex V inhibitor, is added and the resulting OCR is used to derive ATP-linked respiration (by subtracting the oligomycin rate from baseline cellular OCR) and proton leak respiration (by subtracting non-mitochondrial respiration from the oligomycin rate). Next carbonyl cyanide-p-trifluoromethoxyphenyl-hydrazon (FCCP), a protonophore, is added to collapse the inner membrane gradient, allowing the ETC to function at its maximal rate, and maximal respiratory capacity is derived by subtracting non-mitochondrial respiration from the FCCP rate. Lastly, antimycin A and rotenone, inhibitors of complex III and I, are added to shut down ETC function, revealing the non-mitochondrial respiration. Mitochondrial reserve capacity is calculated by subtracting basal respiration from maximal respiratory capacity.

Several measures of mitochondrial respiration, including basal respiration, ATP-linked respiration, proton leak respiration and reserve capacity, were derived by the sequential addition of pharmacological agents to the respiring cells, as diagramed in Figure 1. For each parameter, three repeated rates of oxygen consumption are made over an 18 minute period. First, baseline cellular oxygen consumption is measured, from which basal respiration is derived by subtracting non-mitochondrial respiration. Next oligomycin, an inhibitor of complex V, is added, and the resulting OCR is used to derive ATP-linked respiration (by subtracting the oligomycin rate from baseline cellular OCR) and proton leak respiration (by subtracting non-mitochondrial respiration from the oligomycin rate). Next carbonyl cyanide-p-trifluoromethoxyphenyl-hydrazon (FCCP), a protonophore, is added to collapse the inner membrane gradient, driving the ETC to function to its maximal rate, and maximal respiratory capacity is derived by subtracting non-mitochondrial respiration from the FCCP OCR. Lastly, antimycin A, a complex III inhibitor, and rotenone, a complex I inhibitor, are added to shut down ETC function, revealing the non-mitochondrial respiration. The mitochondrial reserve capacity is calculated by subtracting basal respiration from maximal respiratory capacity.

ECAR is primarily a measure of lactate production and can be equated to the glycolytic rate (i.e., glycolysis), and ECAR is measured simultaneously with OCR in the Seahorse assay. Basal ECAR refers to the ECAR measured before the injection of oligomycin. Glycolytic reserve capacity is calculated by subtracting the basal ECAR from the oligomycin-induced ECAR.

One hour prior to the assay, cells were seeded onto poly-D-lysine coated 96-well XF-PS plates at a density of 1.1×10^5^ cells/well in DMEM XF assay media (unbuffered DMEM supplemented with 11 mM glucose, 2 mM L-glutamax, and 1 mM sodium pyruvate). Cells were plated with at least 4 replicate wells for each treatment group. Titrations were performed to determine the optimal concentrations of oligomycin (1.0 µM), FCCP (0.3 µM), antimycin A (0.3 µM) and rotenone (1.0 µM).

### Redox Challenge

ROS was increased *in vitro* by exposing cells to increasing concentrations of the redox cycling agent, DMNQ (2,3-dimethoxy-1,4-napthoquinone; Sigma-Aldrich, St. Louis, MO, USA), for 1 h prior to the Seahorse assay. DMNQ enters cells and generates both superoxide and hydrogen peroxide similar to levels generated by nicotinamide adenine dinucleotide phosphate oxidase *in vivo* [40]. A 5 mg/mL DMNQ solution was diluted in DMEM XF assay media into 10X stocks and added to cells in an XF-PS plate and incubated for 1 h at 37°C in a non-CO_2_ incubator. The concentrations of DMNQ were optimized as 5 µM, 10 µM, 12.5 µM and 15 µM.

### Inhibition of UCP2

To determine the effects of UCP2 inhibition on mitochondrial respiration in the AD LCLs, we treated the LCLs with genipin, an extract from *Gardenai jasminoides*, and a known UCP2 inhibitor. For these experiments, LCLs were cultured with 50 µM genipin (Sigma-Aldrich) for 24 h prior to the Seahorse assay. Titrations were performed to determine the optimal dose of genipin to alter proton leak respiration without significantly affecting cell viability.

### Immunoblot Analysis

LCLs were lysed using RIPA lysis buffer containing 1% NP40, 0.1% SDS, 1% PMSF, 1% protease inhibitor cocktail and 1% sodium orthovanadate (Santa Cruz, Dallas, TX, USA). Protein concentration was determined using a BCA Protein Assay Kit (BioRad, Hercules, CA, USA), and lysates were prepared with 4X Laemmli Sample Buffer (BioRad) and 5% beta-mercaptoethanol. Samples were boiled for 5 min and cooled on ice for 5 min, and 50 µg of protein per lane was electrophoresed on a 10% polyacrylamide gel (BioRad) and transferred to a 0.45 µM PVDF membrane (Millipore, Billerica, MA, USA). Transfer efficiency was tested by Ponceau S staining (Santa Cruz) of gels. Membranes were probed overnight at 4°C with goat anti-UCP2 (1 µg/ml, R&D Systems, Minneapolis, MN, USA) after blocking with 2% non-fat milk. For detection, the membranes were incubated with donkey anti-goat-HRP (1∶5000, R&D Systems) and the blots were visualized using enhanced chemiluminescence (Thermo Scientific, Pittsburgh, PA, USA) and quantitated using ImageJ (National Institutes of Health, Bethesda, MD, USA). The membrane was stained for total proteins using the Reversible Protein Stain Kit (Pierce, Inc) and the darkest band was quantitated using ImageJ.

### Mitochondrial DNA Copy Number

Relative mitochondrial DNA (mtDNA) copy number, the ratio of the amount of mtDNA to nuclear DNA (nDNA), was compared in the two AD LCL subgroups (n = 7 AD-A; n = 16 AD-N). Total genomic DNA was purified from LCLs using the Gentra Puregene Cell Kit (Quiagen, Germantown, MD, USA), and the concentration of DNA was measured using Nano Drop (Thermo Scientific). Real-time PCR was used to amplify three mitochondrial genes, *ND1, ND4*, and *Cyt B,* and one nuclear gene, *PK*, to assess the relative mtDNA copy numbers, as described in detail [41]. Primers were purchased from IDT (Coralville, IA, USA) and SYBR green mastermix from Applied Biosystems (Carlsbad, CA, USA), and all reactions were run on an ABI 7300 Real-Time PCR system. Relative mtDNA copy number was calculated using the following equation: mtDNA/nDNA = 2^−ΔCt^, where ΔCt = Ct_mito_-Ct_nuclear_.

### Redox Metabolite Measurements

Approximately 5×10^6^ viable cells were pelleted and snap-frozen on dry ice. Samples were stored at −80°C until HPLC quantification of intracellular free reduced glutathione (GSH), oxidized glutathione (GSSG), free reduced cysteine and oxidized cysteine (cystine) [42]. Briefly, thawed cells were lysed by 3 s sonication in 112.5 µl ice-cold PBS followed by the addition of 37.5 µl ice-cold 10% meta-phosphoric acid. This mixture was incubated for 30 min on ice followed by centrifuging for 15 min at 18,000×g at 4°C. The metabolites were eluted using a Shimadzu solvent delivery system (ESA model 580; ESA Inc., Chelmsford, MA) and a reverse-phase C18 column (3 µ, 4.6×150 mm; Shiseido Co., Tokyo, Japan). A 20 µl aliquot of cell extract was directly injected onto the column using an ESA Inc. autosampler (model 507E), and the metabolites were quantified using a model 5200A Coulochem II and CoulArray electrochemical detection system (ESA) equipped with a dual analytical cell (model 5010), a 4-channel analytical cell (model 6210), and a guard cell (model 5020). 3-nitrotyrosine was determined as described [43] with a slight modification of chromatography to optimize retention time for the 3-nitrotyrosine standard. NAD^+^ and NADH were measured as described [44] utilizing a Dionex UltiMate 3000 HPLC-UV system (Dionex Inc., Sunnyvale, CA), C18 Gemini column (5 µ, 100×200 mm; Phenomenex, Torrance, CA) at 254 nm wavelength. Concentrations were calculated from peak areas of standard calibration curves using HPLC software. Results are expressed per protein using BCA Protein Assay Kit (Pierce Inc., Rockford, IL, USA).

### Detection of ROS and Mitochondrial Membrane Potential

CellROX Green (Invitrogen) is a membrane-permeable ROS-sensitive probe that remains non-fluorescent until oxidized by intracellular free radicals. The intensity of CellROX Green fluorescence is proportional to the level of free radical oxidation. LCLs were loaded with 5 µM CellROX Green in culture medium and stained in the dark for 30 min at 37°C. Stained cells were washed and suspended in PBS and analyzed immediately on a BD FACSCalibur (BD Biosciences, San Jose, CA, USA) using 488 nm excitation wavelength with 530/30 nm (FL1) emission filter.

Mitochondrial superoxide was measured using MitoSox Red (Invitrogen), a fluorescent probe targeted to the mitochondria and specific for superoxide. LCLs were loaded with 5 µM MitoSox Red in Hanks Balanced Salt Solution (HBSS) with calcium and magnesium for 30 min at 37°C. Stained cells were washed and suspended in HBSS and analyzed immediately on a BD FACSCalibur using 488 nm excitation wavelength with 585/42 nm (FL2) emission filter.

Mitochondrial membrane potential was measured using JC-1 (Invitrogen), a lipophilic cationic dye that accumulates in mitochondria in a membrane potential-dependent manner. In cells with high mitochondrial membrane potential, JC-1 selectively enters the mitochondria, where it forms aggregates with a high red/green (FL2/FL1) fluorescence intensity. LCLs were loaded with 2 µM JC-1 in culture medium for 15 min at 37°C. Stained cells were washed and suspended in PBS and analyzed immediately on a BD FACSCalibur using 488 nm excitation wavelength with 530/30 nm (FL1) and 585/42 nm (FL2) emission filters.

For each analysis, the fluorescence properties of 10 000 cells were collected, and the data were analyzed using the FCS Express software (De Novo Software, Los Angeles, Calif, USA). Results are expressed as mean fluorescence intensity (MFI) of 10 000 cells.

### Analytic Approach

A mixed-model regression [45] was conducted via SAS version 9.3 (Cary, NC, USA) ‘glmmix’ procedure. The mixed-model allowed data from each AD LCL to be compared to the paired control LCL run on the same plate. The mitochondrial respiratory measurement (or glycolytic parameter) was the response variable with a between-group dichotomous effect (e.g., AD v control) and within-group repeated factor of DMNQ concentration (modeled as a multilevel factor) as well as the interaction between these effects. We present the *overall* difference between the two comparison groups (Group Effect), the overall effect of the DMNQ concentration (DMNQ effect), and the whether the effect of DMNQ concentration was different between the two groups (DMNQ x group interaction). This same analysis was used to analyze the difference in mitochondrial respiratory parameters between each AD subgroup and matched controls. A similar analysis was used to compare mitochondrial respiratory parameters between to the two AD subgroups, although the individual LCLs were not matched across the two AD subgroups. For the analysis of the effect of genipin across subgroups, a within-group dichotomous variable was used to represent genipin exposure and all interactions with DMNQ concentration and AD subgroup were analyzed. For all models, random effects included the intercept and DMNQ. F-tests were used to evaluate significance. Planned post-hoc orthogonal contrasts were used when the interaction was significant. For several interactions, all possible comparisons were statistically significant, in which case the individual comparisons were not reported in the main text but were presented graphically in the figures.

Differences in other measurements (glutathione parameters, fluorescent probes, UCP2 content, mtDNA copy number) between control and AD LCLs and between AD LCL subgroups without DMNQ exposure were analyzed using a similar mixed-effect regression model. For analysis of the DMNQ effect on AD and control LCLs, a general linear model was used to verify the DMNQ effect as these LCLs were not matched. DMNQ was treated as a continuous variable since a dose response effect was expected.

Cluster analysis was conducted using Ward’s technique [46]. Ward’s technique defines the distance between clusters in terms of the between cluster variability to the within cluster variability. By examining the dendogram and several statistics (pseudo F and t^2^), a judgment is made about the number of clusters [47]. Differences in reserve capacity and change in reserve capacity between individual matched pairs of AD and controls were used as variables in the cluster analysis.

## Results

### Mitochondrial Function in AD LCLs with ROS Challenge

**Figure 2:**
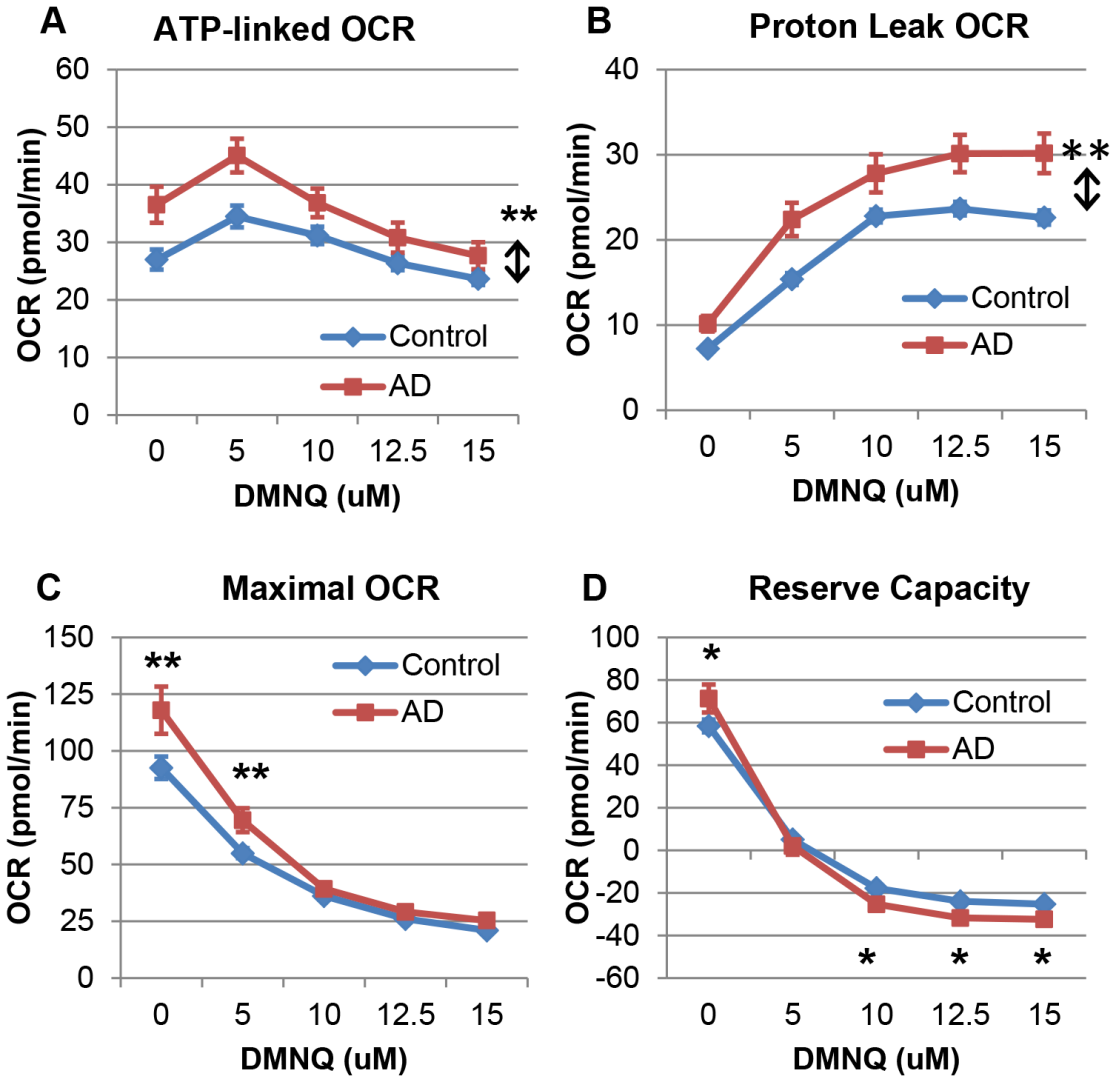
AD LCLs demonstrate differences in mitochondrial function as compared to control LCLs at baseline and after exposure to DMNQ. (**A**) ATP-linked respiration and (**B**) proton leak respiration were overall significantly higher in the AD LCLs, and there was a greater increase in proton leak respiration with DMNQ as compared to control LCLs. (**C**) Maximal respiratory capacity was significantly elevated in the AD LCLs at 0 µM and 5 µM DMNQ compared to control LCLs, and the AD LCLs exhibited a greater decrease in maximal capacity as DMNQ increased as compared to control LCLs. (**D**) Reserve capacity was significantly elevated in the AD LCLs at baseline, and it decreased with DMNQ so that it was significantly lower than control LCLs at 10–15 µM DMNQ. *p<0.001; **p<0.0001; ↕ indicates an overall statistical difference between LCL groups.

ATP-linked respiration was overall higher for AD LCLs as compared to control LCLs [F(1,776) = 79.43, p<0.0001] (Figure 2A). ATP-linked respiration changed significantly as DMNQ increased [F(4,96) = 39.11, p<0.0001] such that it increased to a peak at 5 µM DMNQ and then slowly decreased following this peak. The change in ATP-linked respiration with increasing DMNQ was not significantly different between the two LCLs groups.

Proton leak respiration was overall higher in AD LCLs [F(1,776) = 197.08, p<0.0001] (Figure 2B) and significantly increased as DMNQ increased [F(4,96) = 176.89, p<0.0001]. This increase was significantly greater for AD LCLs [F(4,776) = 2.81, p<0.05], and proton leak respiration was significantly different between the two groups for all DMNQ concentrations.

Maximal respiratory capacity was overall significantly higher in AD LCLs [F(1,776) = 82.65, p<0.0001] and decreased as DMNQ increased [F(4,96) = 77.46, p<0.0001]. This decrease was greater for AD LCLs as compared to control LCLs [F(4,776) = 16.10, p<0.0001] (Figure 2C). This greater decrease in AD LCLs resulted in the maximal respiratory capacity being significantly greater in the AD LCLs as compared to control LCLs at 0 µM DMNQ [t(776) = 10.43, p<0.0001] and 5 µM DMNQ [t(776) = 5.58, p<0.0001] but not at the higher DMNQ concentrations.

Reserve capacity was overall not different between the AD and control LCL groups but demonstrated a significant interaction between the groups. As DMNQ increased, reserve capacity decreased [F(4,96) = 126.72, p<0.0001] with this decrease significantly greater for AD LCLs [F(4,776) = 28.48, p<0.0001]. Reserve capacity of AD LCLs started out significantly higher than control LCLs at 0 µM DMNQ [t(776) = 8.21, p<0.0001], but then dropped sharply to become non-significantly different than control LCLs at 5 µM DMNQ and then significantly lower than control LCLs at higher DMNQ concentrations [10 µM DMNQ t(776) = 3.42, p<0.001; 12.5 µM DMNQ t(776) = 4.50, p<0.001; 15 µM DMNQ t(776) = 4.15, p<0.001] (Figure 2D).

### Defining Subgroups of AD LCLs

**Figure 3:**
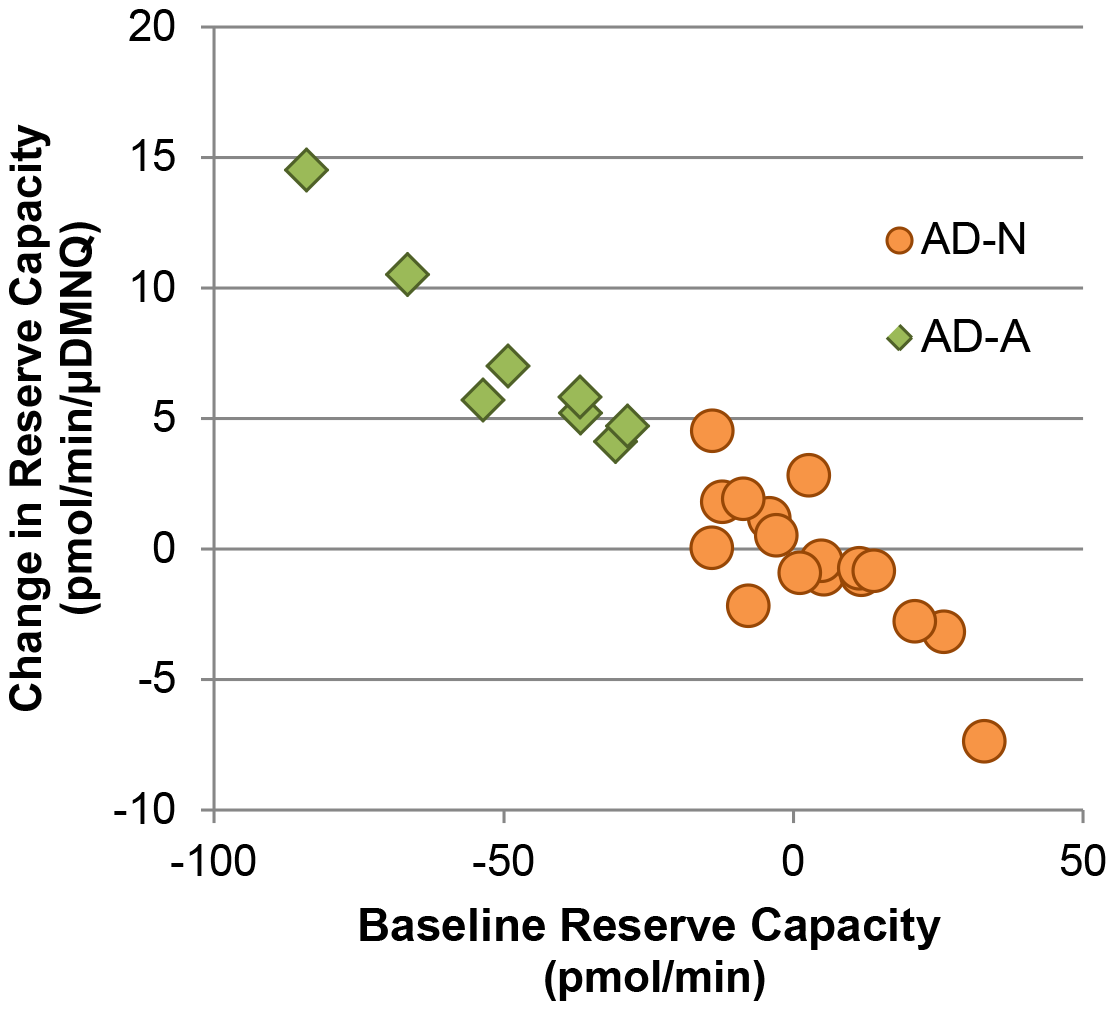
The AD LCLs cluster into two subgroups. The difference in baseline reserve capacity between control and AD pairs was plotted against the difference in the change in reserve capacity (from 0 to 10 µM DMNQ) between control and AD pairs. The AD-A subgroup (green diamonds) exhibited greater differences in baseline reserve capacity and change in reserve capacity as compared to the paired control LCLs, whereas the AD-N subgroup (orange circles) exhibited reserve capacity parameters more similar to the paired control LCLs.

Since AD and control LCLs differed markedly in the changes in reserve capacity with DMNQ challenge, we examined the changes in reserve capacity to differentiate AD LCL subgroups. Since the decrease in reserve capacity bottomed out at 10 µM DMNQ, the slope of the change in reserve capacity from 0 to 10 µM DMNQ was calculated and entered into a cluster analysis along with the baseline reserve capacity. The cluster analysis divided the LCLs into two groups: AD-N (n = 17) and AD-A (n = 8) [pseudo t^2^ = 58.5] (See Figure 3). The dendogram (not shown) demonstrated clear differences between these groups.

### Mitochondrial Function in AD LCLs Subgroups with ROS Challenge

To better understand the differences between the two AD LCL subgroups, we compared the AD LCLs to their paired control LCLs within each subgroup as well as compared the two AD subgroups to each other.

#### AD-N v control LCLs

**Figure 4:**
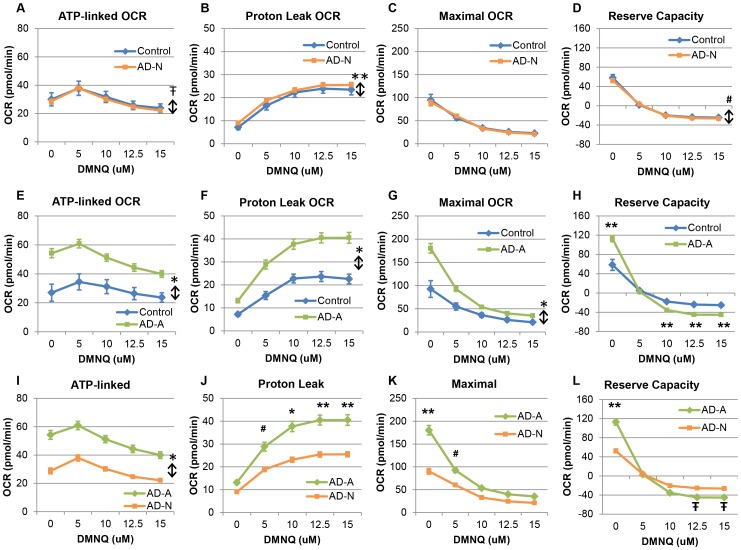
Mitochondrial respiratory parameters and responses to DMNQ differ in two AD LCL subgroups. Overall, the AD-N subgroup (A–D) demonstrates similar mitochondrial responses as the control LCLs while the AD-A subgroup (E–H) parallels the differences between the AD and control LCLs found in the overall analysis. For the AD-N subgroup (**A**) ATP-linked respiration and (**D**) reserve capacity were overall slightly but significantly lower in the AD-N LCLs while (**B**) proton leak respiration was overall slightly but significantly higher in the AD-N LCLs, and (**C**) maximal respiratory capacity was not different in the AD-N LCLs as compared to controls. For the AD-A subgroup, (**E**) ATP-linked respiration, (**F**) proton leak respiration and (**G**) maximal respiratory capacity were overall markedly higher for AD-A LCLs as compared to control LCLs. (**H**) Reserve capacity was significantly greater for the AD-A LCLs as compared to control LCLs at baseline but decreased such that it was significantly lower than controls at 10–15 µM DMNQ. (**I**) ATP-linked respiration was overall markedly higher for AD-A LCLs as compared to AD-N LCLs. (**J**) Proton leak respiration was significantly higher in the AD-A LCLs as compared to the AD-N LCLs at 5–15 µM DMNQ. (**K**) Maximal respiratory capacity was significantly higher for AD-A LCLs as compared to AD-N LCLs at baseline and 5 µM DMNQ. (**L**) Reserve capacity was significantly greater for the AD-A LCLs at baseline but decreased so that it was significantly lower for the AD-A LCLs as compared to the AD-N LCLs at 12.5 and 15 µM DMNQ. *p<0.001; **p<0.0001; # p<0.01; 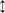 p<0.05; ↕ indicates an overall statistical difference between LCL groups.

ATP-linked respiration was slightly but significantly lower in the AD-N as compared to control LCLs [F(1,516) = 4.36, p<0.05]. While ATP-linked respiration changed significantly as DMNQ increased [F(4,64) = 22.34, p<0.0001], this change was not different between groups (Figure 4A). Like the overall analysis, ATP-linked respiration increased to a peak at 5 µM and then decreased after this peak.

Overall proton leak respiration was slightly but significantly higher in the AD-N as compared to the control LCLs [F(1,516) = 16.52, p<0.0001] (Figure 4B). Proton leak respiration increased as DMNQ increased [F(4,64) = 129.58, p<0.0001] but this change was not significantly different between the two groups.

Maximal respiratory capacity significantly decreased as DMNQ increased [F(4,64) = 48.00, p<0.0001] but neither overall maximal respiratory capacity nor the change in maximal respiratory capacity with increasing DMNQ were significantly different across the two LCL groups (Figure 4C).

Overall reserve capacity was slightly but significantly lower in the AD-N as compared to the control LCLs [F(1,516) = 7.49, p<0.01]. Reserve capacity significantly decreased as DMNQ increased [F(4,64) = 84.46, p<0.0001] with this change significantly different between the two LCL groups [F(4,516) = 2.80, p<0.05]. This interaction occurred because reserve capacity was slightly but significantly lower for AD-N as compared to control LCLs at baseline but not when challenged with DMNQ [t(516) = 3.76, p<0.001] (Figure 4D).

#### AD-A v control LCLs

Overall, ATP-linked respiration was markedly and significantly higher for AD-A LCLs [F(1,255) = 454.32, p<0.001] (Figure 4E). ATP-linked respiration significantly changed as DMNQ increased [F(4,28) = 17.20, p<0.0001] with this change significantly different for AD-A LCLs as compared to the control LCLs [F(4,255) = 2.92, p<0.05]. For both the AD-A and control LCLs, ATP-linked respiration increased to a peak at 5 µM and then decreased after this peak. However, the difference in ATP-linked respiration between the AD-A and control LCLs was greater at lower DMNQ concentrations than higher DMNQ concentrations, although ATP-linked respiration was significantly greater in the AD-A LCLs as compared to the control LCLs at each individual DMNQ concentration.

Overall, proton leak respiration was markedly and significantly higher for AD-A LCLs [F(1,255) = 479.14, p<0.001] (Figure 4F). Proton leak respiration significantly increased as DMNQ increased [F(4,28) = 84.19, p<0.0001] with this increase significantly greater for AD-A LCLs as compared to the control LCLs [F(4,255) = 11.59, p<0.0001].

Overall, maximal respiratory capacity was markedly higher for AD-A LCLs [F(1,255) = 378.43, p<0.001] (Figure 4G). Maximal respiratory capacity significantly decreased as DMNQ increased [F(4,28) = 43.08, p<0.0001] with this decrease significantly greater for AD-A LCLs as compared to the control LCLs [F(4,255) = 65.04, p<0.0001] such that the difference in maximal respiratory capacity between the AD-A and control LCLs was much greater at 0 µM DMNQ as compared to 15 µM DMNQ (although the difference between groups remained significant at all concentrations of DMNQ).

Overall, reserve capacity was not markedly different between the AD-A and control LCLs but demonstrated a significant interaction between groups as DMNQ increased. Reserve capacity significantly decreased as DMNQ increased [F(4,28) = 67.71, p<0.0001] with this decrease significantly more marked for AD-A LCLs as compared to the control LCLs [F(4,255) = 115.69, p<0.0001]. Reserve capacity was significantly greater for the AD-A LCLs as compared to control LCLs at baseline (i.e., 0 µM) [t(255) = 18.51, p<0.0001] but sharply decreased as DMNQ increased such that it was significantly lower for the AD-A LCLs as compared to the control LCLs at 10 µM [t(255) = 5.59, p<0.0001], 12.5 µM [t(255) = 6.84, p<0.0001] and 15 µM DMNQ [t(255) = 6.49, p<0.0001] (Figure 4G).

#### AD-A v AD-N LCLs

Overall, ATP-linked respiration was markedly higher for AD-A LCLs as compared to AD-N LCLs [F(1,349) = 16.01, p<0.001] (Figure 4I). ATP-linked respiration changed significantly as DMNQ increased [F(4,91) = 30.59, p<0.0001] but this change was not different between the two AD LCL subgroups.

Overall, proton leak respiration was markedly higher for AD-A LCLs as compared to AD-N LCLs [F(1,349) = 11.49, p<0.001] (Figure 4J). Proton leak respiration significantly increased as DMNQ increased [F(4,91) = 159.33, p<0.0001] with this increase significantly greater for AD-A LCLs as compared to the AD-N LCLs [F(4,349) = 10.15, p<0.0001]. This interaction was due to the fact that proton leak respiration was not significantly different between the two AD subgroups at baseline but became significantly higher when DMNQ was added [5 µM t(349) = 3.12, p<0.01; 10 µM t(349) = 3.76, p<0.001; 12.5 µM t(349) = 3.98, p<0.0001; 15 µM t(349) = 3.95, p<0.0001].

Overall, maximal respiratory capacity was markedly higher for AD-A LCLs as compared to AD-N LCLs [F(1,349) = 15.21, p<0.001] (Figure 4K). Maximal capacity significantly decreased as DMNQ increased [F(4,91) = 100.32, p<0.0001] with this decrease significantly greater for AD-A LCLs as compared to the AD-N LCLs [F(4,349) = 13.21, p<0.0001] such that the difference in maximal capacity between the AD-A and AD-N LCLs was significant at lower DMNQ concentrations [0 µM t(349) = 7.59, p<0.0001; 5 µM t(349) = 2.31, p<0.01] but not at higher DMNQ concentrations.

Overall, reserve capacity was not markedly different between the AD-A and AD-N LCLs but demonstrated a significant interaction between groups as DMNQ increased. Overall, reserve capacity significantly decreased as DMNQ increased [F(4,91) = 146.84, p<0.0001] with this decrease significantly more marked for AD-A LCLs as compared to the AD-N LCLs [F(4,349) = 17.16, p<0.0001]. Reserve capacity was significantly greater for the AD-A LCLs at baseline (i.e., 0 µM) [t(349) = 7.42, p<0.0001] but sharply decreased so that it was significantly lower for the AD-A LCLs as compared to the AD-N LCLs at 12.5 µM [t(349) = 2.36, p<0.02] and 15 µM [t(255) = 2.19, p<0.02] DMNQ (Figure 4L).

### Extracellular Acidification Rate

**Figure 5:**
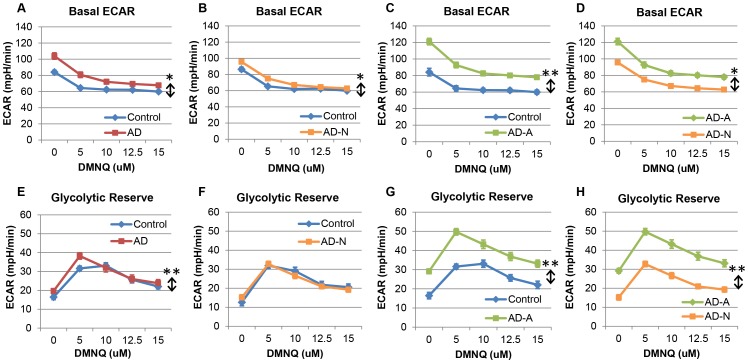
Extracellular acidification rate (ECAR) differs in AD-A and AD-N LCLs. Basal ECAR was overall significantly higher, and the decrease in ECAR with DMNQ was also greater for the (**A**) AD LCLs as a whole, (**B**) the AD-N LCLs and (**C**) the AD-A LCLs as compared to matched controls. (**D**) The AD-A LCLs had an overall significantly higher basal ECAR than the AD-N LCLs. (**E**) Glycolytic reserve capacity was overall higher in the AD LCLs as a whole compared to the control LCLs, but was not different between (**F**) AD-N and controls. (**G**) The AD-A LCLs exhibited overall higher glycolytic reserve capacity as compared to the control LCLs and compared to the (**H**) AD-N LCLs. *p<0.001; **p<0.0001; ↕ indicates an overall statistical difference between LCL groups.

Basal ECAR was overall significantly higher in the AD LCLs as compared to the control LCLs [F(1,835) = 226.24, p<0.001] and decreased as DMNQ concentration increased [F(4,96) = 123.07, p<0.0001] with a greater decrease for the AD LCLs as compared to the control LCLs [F(4,835) = 9.01, p<0.001] (Figure 5A). The AD-N LCLs also demonstrated higher basal ECAR than the control LCLs [F(1,569) = 49.97, p<0.001] and the significant decrease in ECAR with increasing DMNQ concentrations [F(4,64) = 92.55, p<0.0001] was greater in magnitude for the AD-N LCLs as compared to the control LCLs [F(4,569) = 3.59, p<0.01] (Figure 5B). The same phenomenon was seen for the AD-A LCLs but with a much greater difference between the AD-A and control LCLs as compared to the difference between the AD-N and control LCLs (Figure 5C). Indeed, basal ECAR was significantly higher in the AD-A LCLs as compared to the control LCLs [F(1,261) = 517.89, p<0.0001], and the significant decrease in basal ECAR with increasing DMNQ concentrations [F(4,28) = 32.22, p<0.0001] was greater for the AD-A LCLs as compared to the control LCLs [F(4,261) = 12.30, p<0.0001]. When the two AD subgroups were compared, AD-A LCLs were found to have a significantly higher basal ECAR than the AD-N LCLs [F(1,361) = 6.83, p<0.01], and the significant decrease in ECAR with increasing DMNQ concentrations [F(4,92) = 120.02, p<0.0001] was significantly greater in magnitude for AD-A LCLs as compared to AD-N LCLs [F(4,361) = 2.37, p = 0.05] (Figure 5D). These data demonstrate that, in general, AD LCLs are more dependent on glycolysis for energy production with this dependency being particularly significant for the AD-A LCLs as compared to the AD-N LCLs.

Overall, glycolytic reserve capacity was found to be higher in the AD LCLs as compared to the control LCLs [F(1,835) = 56.17, p<0.0001] (Figure 5E). Glycolytic reserve capacity was found to change significantly as DMNQ increased [F(4,96) = 60.29, p<0.0001] peaking at 5 µM DMNQ and then decreasing at higher DMNQ concentrations. There was a significant DMNQ by group interaction [F(4,835) = 3.0, p = 0.02] due to the fact that glycolytic reserve capacity was greater for the AD LCLs as compared to the control LCLs at lower DMNQ concentrations but decreased to become more alike as DMNQ concentration increased. When we examined the two AD subgroups separately, we found that the glycolytic reserve capacity for the AD-N LCLs was not significantly different than the control LCLs (Figure 5F), and the significant change in glycolytic reserve capacity with increasing DMNQ [F(4,64) = 38.37, p<0.0001] was not different between groups. However, the AD-A LCLs did demonstrate a significantly higher glycolytic reserve capacity as compared to the control LCLs [F(1,261) = 294.14, p<0.0001] (Figure 5G). Glycolytic reserve capacity changed significantly as DMNQ increased [F(4,28) = 20.53, p<0.0001] with this change significantly different across the two LCL groups [F(4,261) = 3.54, p<0.01]. Glycolytic reserve capacity was greater for the AD-A LCLs as compared to the control LCLs at lower DMNQ concentrations but decreased to become more alike as DMNQ concentration increased. Comparing the two LCL groups revealed that the AD-A LCLs exhibited a significantly higher glycolytic reserve capacity as compared to the AD-N LCLs [F(1,361) = 15.29, p<0.0001] (Figure 5H). Glycolytic reserve capacity changed significantly as DMNQ increased [F(4,92) = 35.86, p<0.0001] although this pattern of change was not significantly different across the two groups.

### Inhibition of UCP2 Affects AD-N and AD-A LCLs Differently

In order to determine the differential ability of the AD LCL subgroups to adapt to intramitochondrial oxidative stress at the inner mitochondrial membrane, we used genipin to inhibit UCP2, the key protein on the inner mitochondrial membrane which regulates proton leak to reduce ETC generated oxidative stress. For this set of experiments, we used only two concentrations of DMNQ, 0 µM and 10 µM.

**Figure 6:**
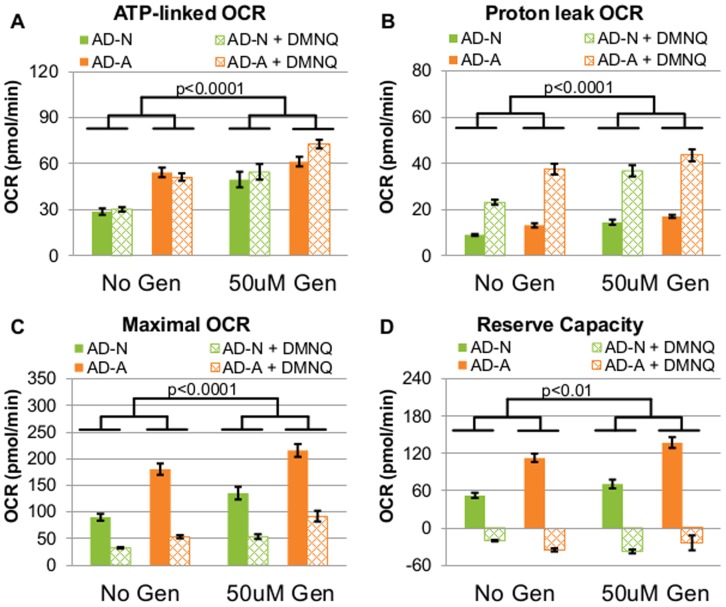
Inhibition of UCP2 with Genipin affects AD-A and AD-N LCLs differently. (**A**) ATP-linked respiration was overall higher in LCLs exposed to genipin compared to unexposed LCLs, and cells exposed to genipin exhibited a greater increase in ATP-linked respiration with DMNQ compared to cells unexposed to genipin. (**B**) Proton leak respiration was overall higher in the LCLs exposed to genipin as compared to the unexposed LCLs, and pretreatment with genipin resulted in a greater increase in proton leak respiration for the AD-N LCLs as compared to the AD-A LCLs. LCLs exposed to genipin had a greater increase in proton leak respiration with DMNQ compared to cells unexposed to genipin. (**C**) Maximal respiratory capacity was overall higher in the LCLs exposed to genipin than the LCLs not exposed to genipin, and the decrease in maximal capacity with DMNQ was greater for the genipin treated LCLs compared to the genipin unexposed LCLs. (**D**) Reserve capacity was overall higher in the LCLs exposed to genipin as compared to the unexposed LCLs, and the increase in reserve capacity with genipin was greater for the AD-A LCLs than the AD-N LCLs. The decrease in reserve capacity with DMNQ was significantly greater for the genipin treated LCLs as compared to the genipin unexposed cells.

Overall, LCLs exposed to genipin exhibited higher ATP-linked respiration than unexposed LCLs [F(1,379) = 73.75, p<0.0001] (Figure 6A). ATP-linked respiration was also overall higher for the AD-A than the AD-N LCLs [F(1,379) = 4.43, p<0.05]. Interestingly, there was a DMNQ by genipin interaction [F(1,379) = 4.33, p<0.05] such that ATP-linked respiration did not increase with DMNQ for the LCLs unexposed to genipin, but it increased significantly with DMNQ in the LCLs exposed to genipin [t(379) = 7.98, p<0.0001].

Overall, proton leak respiration was greater for the LCLs exposed to genipin as compared to the unexposed LCLs [F(1,379) = 74.50, p<0.0001] (Figure 6B). It was also higher in the AD-A LCLs compared to the AD-N LCLs [F(1,379) = 4.34, p<0.05] and in the LCLs exposed to DMNQ compared to the LCLs not treated with DMNQ [F(1,23) = 89.02, p<0.0001]. There was a DMNQ by genipin interaction [F(1,379) = 9.70, p<0.01] because the increase in proton leak respiration associated with DMNQ was greater for the LCLs exposed to genipin as compared to those unexposed to genipin. Genipin also resulted in a more significant increase in proton leak respiration for the AD-N LCLs [t(379) = 8.90, p<0.0001] as compared to the AD-A LCLs [t(416) = 4.05, p<0.0001; F(1,379) = 4.85, p<0.05] demonstrating that the AD-N LCLs had a significantly greater ability to adapt to inhibition of the UCP2 protein by increasing proton leak across the inner mitochondrial membrane.

Overall, the LCLs exposed to genipin exhibited higher maximal respiratory capacity than the LCLs not exposed to genipin [F(1,379) = 76.65, p<0.0001] (Figure 6C). Maximal respiratory capacity was also higher for AD-A LCLs as compared to AD-N LCLs [F(1,379) = 11.92, p<0.001] and for the LCLs not exposed to DMNQ as compared to pretreatment with 10 µM DMNQ [F(1,23) = 70.84, p<0.0001]. There was a DMNQ by genipin interaction [F(1,379) = 9.39, p<0.01] such that the decrease in maximal respiratory capacity with DMNQ was significantly greater for the genipin treated LCLs as compared to the genipin unexposed cells.

Overall, reserve capacity was greater for the LCLs exposed to genipin as compared to the unexposed LCLs [F(1,379) = 8.37, p<0.01] (Figure 6D). Reserve capacity was also greater in the AD-A LCLs as compared to the AD-N LCLs [F(1,379) = 8.17, p<0.01] and for LCLs not exposed to DMNQ as compared to pretreatment with 10 µM DMNQ [F(1,23) = 114.49, p<0.0001]. There was a DMNQ by subgroup interaction [F(1,379) = 10.48, p = 0.001] such that reserve capacity decreased more significantly with DMNQ for the AD-A group than the AD-N group. There was also a DMNQ by genipin interaction [F(1,379) = 19.02, p<0.0001] such that the decrease in reserve capacity with DMNQ was significantly greater for the genipin treated LCLs as compared to the genipin unexposed cells. Lastly, and most interestingly, there was a genipin by LCL subgroup interaction [F(1,379) = 11.78, p<0.001] such that reserve capacity changed less for the AD-N LCLs exposed to genipin [t(379) = 1.95, p = 0.05] as compared to the AD-A LCLs exposed to genipin [t(379) = 3.99, p<0.0001], indicating that the abnormal elevation in reserve capacity seen in the AD-A LCLs was further exacerbated when exposed to genipin.

### Uncoupling Protein 2 Content

**Figure 7:**
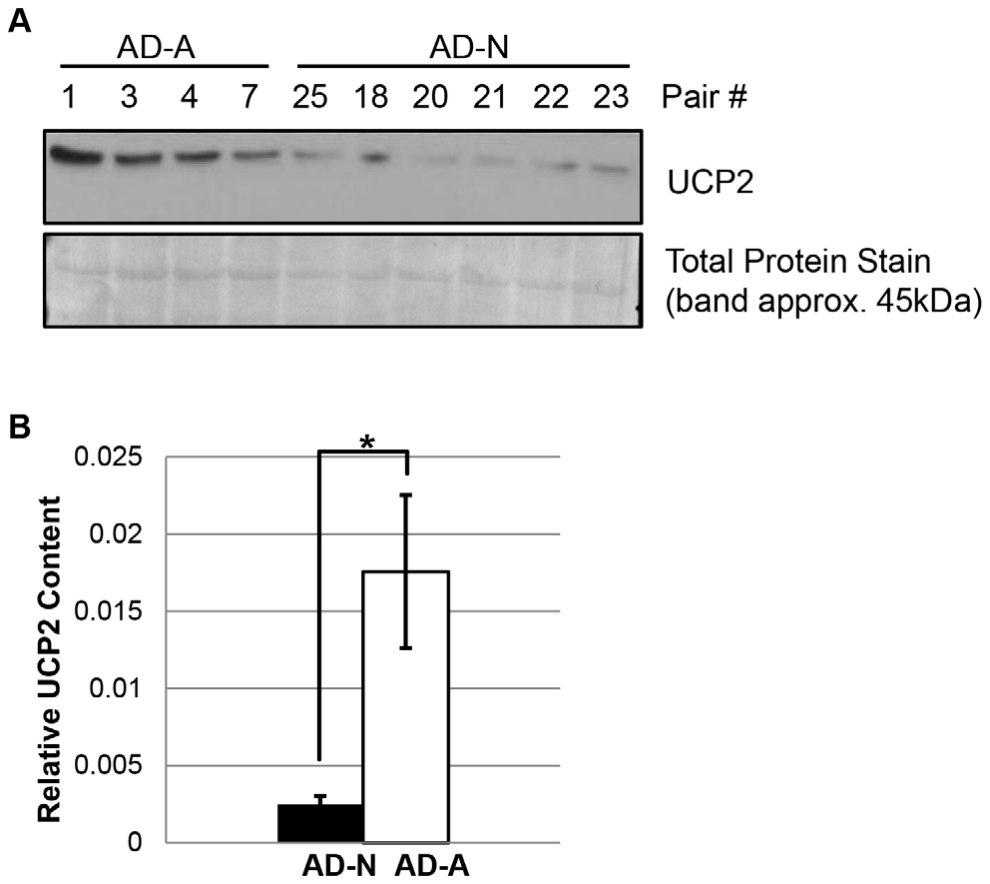
UCP2 content is higher in the AD-A LCL subgroup. (**A**) Immunoblot analysis of UCP2. Cell lysates from AD-N LCLs (N = 4) and AD-A LCLs (N = 6) were analyzed for UCP2 content. A total protein stain served as the loading control. The molecular weight of UCP2 was confirmed using molecular mass markers. (**B**) Quantitation of band densities demonstrates the significantly higher UCP2 content in AD-A LCLs as compared to AD-N LCLs. *p<0.01.

Uncoupling Protein 2 (UCP2) is one of the key regulators of proton leak respiration. Since proton leak respiration is one of the major differences in respiratory parameters between the two AD LCL subgroups, we measured UCP2 content by western blots in a subset of LCLs from both the AD-A (N = 4) and AD-N (N = 6) subgroups to determine whether UCP2 protein content differed between the two AD subgroups at baseline (i.e., without exposure to DMNQ). As shown in Figure 7, the AD-A LCLs were found to have a significantly higher UCP2 protein content as compared to the AD-N LCLs [F(1,8) = 14.51, p<0.01].

### Mitochondrial DNA Copy Number

**Figure 8:**
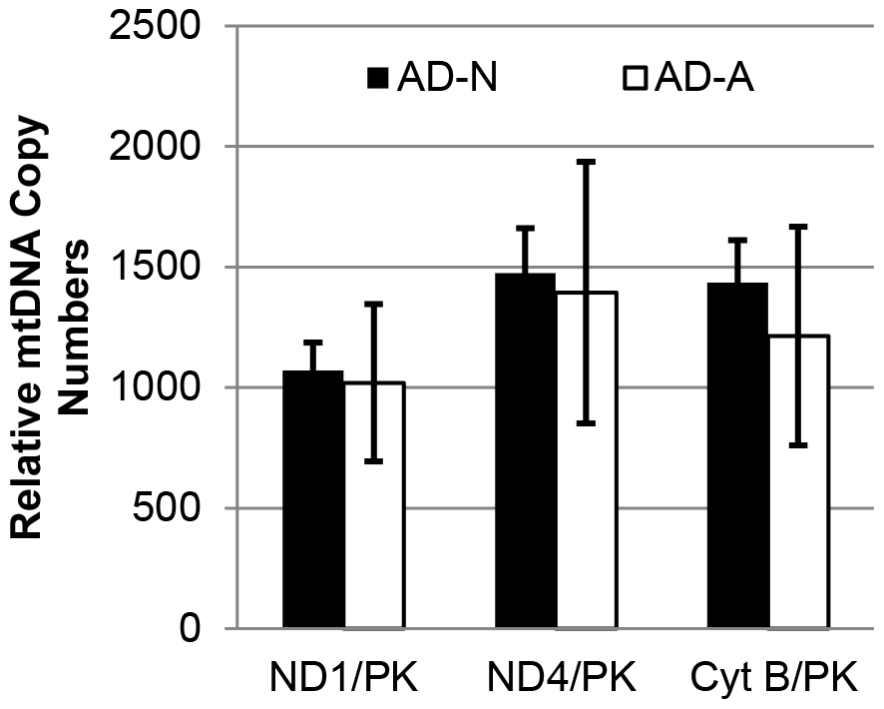
Mitochondrial DNA copy number does not differ between AD LCL subgroups. Relative copy numbers of the mitochondrial genes *ND1*, *ND4*, and *Cyt B* were assessed by normalization with the nuclear gene *PK*. No significant differences were found between two AD LCL subgroups.

To determine whether the number of mitochondria per cell could account for the differences in the respiratory parameters between the two AD LCL subgroups, we measured mtDNA copy number by calculating the ratio of 3 mitochondria genes, including *ND1*, *ND4* and *Cyt B,* to the nuclear gene, *PK*. As shown in Figure 8, the mtDNA copy number was not different between the two AD LCL subgroups; thus, the distinct respiratory parameters observed in the two AD LCL subgroups are not due to differences in mitochondrial number between the groups.

### Intracellular Redox Metabolites and Oxidants in LCLs

**Figure 9:**
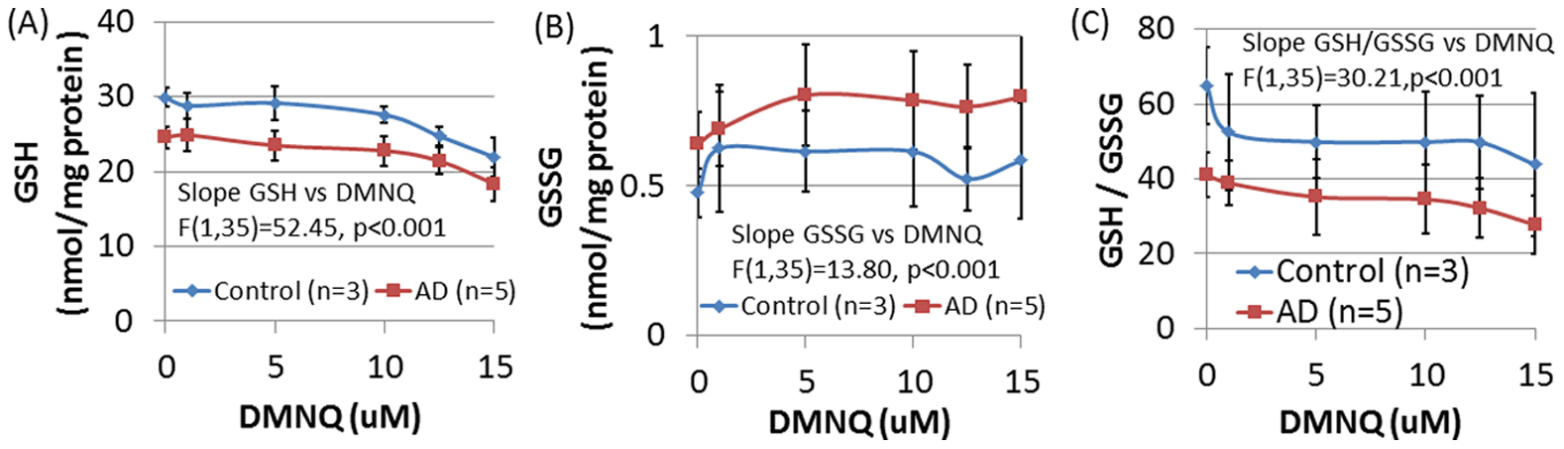
DMNQ exposure alters the glutathione redox status of LCLs. The change in intracellular (**A**) reduced glutathione (GSH), (**B**) oxidized glutathione (GSSG) and (**C**) the reduced-to-oxidized glutathione ratio (GSH/GSSG) with increased intracellular oxidative stress was measured in control (n = 3) and AD (n = 5) LCLs treated with indicated concentrations of the redox cycling agent DMNQ for 1 h. Results are expressed per mg protein. Overall DMNQ significantly reduces GSH and GSH/GSSH and increases GSSG.

To verify that ROS production by DMNQ affects glutathione redox balance, glutathione concentrations were measured in 3 control and 5 AD LCLs at DMNQ concentrations of 0, 1, 5, 10, 12.5 and 15 µM. DMNQ significantly decreased GSH [F(1,35) = 52.45, p<0.001] and GSH/GSSG [F(1,35) = 30.21, p<0.001] and increased GSSG [F(1,35) = 13.80, p<0.001] in a linear fashion (See Figure 9). These changes were not different across groups.

**Figure 10:**
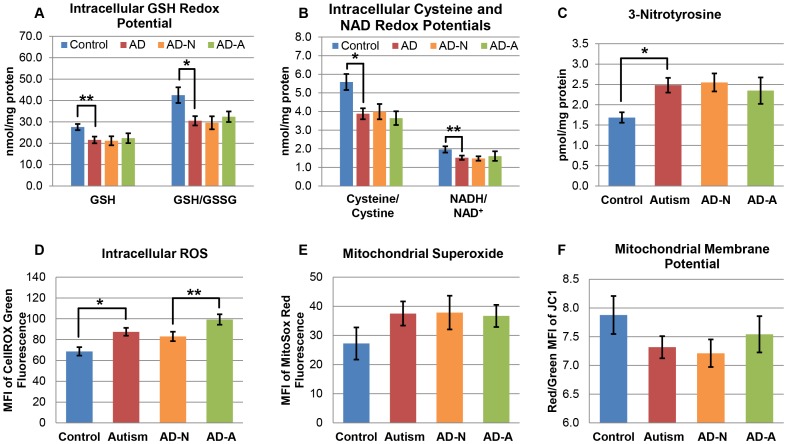
AD LCLs exhibit a more oxidized redox state and increased production of ROS. (**A**) Reduced glutathione (GSH) and the reduced-to-oxidized glutathione ratio (GSH/GSSG) were both significantly lower in AD as compared to control LCLs. (**B**) The ratio of reduced cysteine to oxidized cystine was significantly lower in the AD LCLs as compared to the control LCLs. The NADH/NAD^+^ ratio was also significantly lower in the AD LCLs as compared to control LCLs. The data is presented as the NADH/NAD^+^ ratio x 10 for clarity. (**C**) 3-nitrotyrosine was significantly higher in the AD LCLs as compared to the control LCLs. (**D**) Intracellular ROS was measured by CellRox Green fluorescence, and the AD LCLs demonstrated significantly higher levels of intracellular ROS as compared to control LCLs. Furthermore, the AD-A LCLs demonstrated higher levels of intracellular ROS as compared to the AD-N LCLs. (**E**) Mitochondrial superoxide was measured using MitoSox Red fluorescence, and (**F**) mitochondrial membrane potential was measured using JC-1 fluorescence in the AD and control LCLs. There were no significant differences in either mitochondrial superoxide or mitochondrial membrane potential between any of the LCL groups. *p<0.01; **p<0.05.

We compared the redox status in the AD and control LCLs by measuring three separate redox couples (Figure 10). AD LCLs demonstrated decreased intracellular GSH [F(1,35) = 6.33, p<0.05] and GSH/GSSG ratio [F(1,35) = 9.02, p<0.01] but no difference in GSSG as compared to control LCLs (Figure 10A). The ratio of reduced cysteine to oxidized cystine was lower in the AD LCLs as compared to the control LCLs [F(1,35) = 11.15, p<0.01] (Figure 10B) although intracellular cysteine and cystine concentrations were not significantly different. The ratio of reduced NADH to oxidized NAD^+^ was also significantly lower in the AD LCLs as compared to control LCLs [F(1,35) = 4.50, p<0.05] (Figure 10B) although intracellular NADH and NAD^+^ concentrations were not significantly different. 3-nitrotyrosine, a marker of protein oxidation indicative of chronic oxidative stress, was significantly higher in the AD LCLs as compared to the control LCLs [F(f(1,35) = 9.09, p<0.01] (Figure 10C). The AD-A LCLs did not demonstrate significant differences in any of these intracellular redox markers as compared to the AD-N LCLs. The more oxidized state of three redox couples demonstrates the significantly more oxidized microenvironment of the AD LCLs as compared to the control LCLs.

At baseline (i.e., without any DMNQ challenge) AD LCLs demonstrated significantly higher intracellular ROS as compared to control LCLs [F(1,32) = 9.21, p<0.01]. Furthermore, the AD-A LCLs demonstrated higher intracellular ROS as compared to the AD-N LCLs [F(1,20) = 5.97, p<0.05] (Figure 10D). There were no significant differences in mitochondrial superoxide or mitochondrial membrane potential between the AD and control LCLs or between the AD-A and AD-N subgroups (Figure 10E–F).

## Discussion

This study examined mitochondrial respiratory function in lymphoblastoid cells derived from children with AD at baseline and after exposure to an agent that increased ROS *in vitro*. Here, for the first time, we show that LCLs derived from children with AD exhibit significant abnormalities in mitochondrial respiration before and after exposure to increasing ROS. Specifically, we demonstrate higher ATP-linked and proton leak respiration, maximal respiratory capacity and reserve capacity at baseline and an atypical increase in proton leak respiration along with a sharp drop in both maximal respiratory and reserve capacity in the AD LCLs as compared to the control LCLs with exposure to increasing ROS. By examining reserve capacity we then further demonstrated that these atypical responses were driven by a subset that comprised 32% of the AD LCLs. This subgroup also demonstrated a higher rate of glycolysis and glycolytic reserve as well as increased ROS production. Furthermore, this subgroup exhibited an increased UCP2 content, which, when inhibited with genipin, exacerbated the abnormal respiratory parameters, particularly, increased reserve capacity. Overall, this study suggests that a subset of children with AD may have significant physiological abnormalities in mitochondrial function that results in a vulnerability to oxidative stress such that exposure to ROS induces mitochondrial dysfunction. This evidence provides important insight into the potential pathophysiological mechanisms associated with AD and potential strategies for treatment.

### Differences in Reserve Capacity Depletion in AD LCL Subgroups with ROS Exposure

The reserve capacity at baseline and the change in response to increasing ROS was used to divide the AD LCLs into normal (AD-N) and abnormal (AD-A) subgroups. Reserve capacity was significantly elevated at baseline in the AD-A subgroup relative to both controls and to AD-N LCLs, which likely represents a compensatory adaptive response to the chronic elevations in ROS (demonstrated in the AD-A subgroup using the fluorescent probe CellROX green). Because we demonstrated that that mitochondrial copy numbers were not different in the two AD subgroups, the compensatory response that leads to increased reserve capacity in the AD-A subgroup was not likely due to differences in the number of mitochondria per cell but more likely due to either up-regulation of ETC complexes or to regulation of substrate supply and allosteric regulation of key metabolic enzymes [34], [48].

Despite the elevated reserve capacity at baseline, exposure to ROS resulted in a more precipitous decrease in reserve capacity in the AD-A LCLs as compared to the AD-N LCLs. This is significant since reduced reserve capacity is linked to several diseases such as aging [33], heart disease [34] and neurodegenerative disorders [35], [36]. Reserve capacity is depleted when the mitochondria function at their maximal capacity, and depletion of reserve capacity renders the cell unable to meet any additional ATP demand. Reserve capacity depletion has been shown to result in cell death in several cell types under conditions of oxidative stress including cardiomyocytes [37] and endothelial cells [40], and in neurons during glutamate toxicity or ETC inhibition [36], [49].

Overall proton leak respiration was slightly but significantly higher in AD-N LCLs as compared to control LCLs. This is not surprising as several indices of oxidative stress indicate that the AD-N LCLs have a more oxidized microenvironment as compared to control LCLs. As a result of this slight increase in proton leak respiration, the reserve capacity was slightly reduced in the AD-N LCLs as compared to control LCLs. In contrast to the mild differences in respiratory parameters between the AD-N and control LCLs, the differences in respiratory parameters between the AD-A LCL subgroup and both control and the AD-N LCL subgroup are particularly striking. First, the increased proton leak respiration in the AD-A LCLs compared to control LCLs was much more marked at baseline and became exaggerated as DMNQ increased. Second, the differences in reserve capacity were much more marked with a significantly higher reserve capacity at baseline for the AD-A LCL subgroup (as compared to the control LCLs) with a significant decrease in reserve capacity with increasing DMNQ concentrations. Third, unlike the AD-N LCL subgroup, the AD-A LCL subgroup demonstrated significant elevations in ATP-linked respiration and maximal respiratory capacity at baseline with this difference diminishing as DMNQ increased. These differences were also seen when comparing the AD-A and AD-N subgroup, demonstrating that the AD-A LCLs represent a distinct subgroup of LCLs with an atypical mitochondrial response to chronic and acute increases in intracellular ROS.

### Normal Adaptive and Maladaptive Responses to a more Oxidized Intracellular Microenvironment

**Figure 11:**
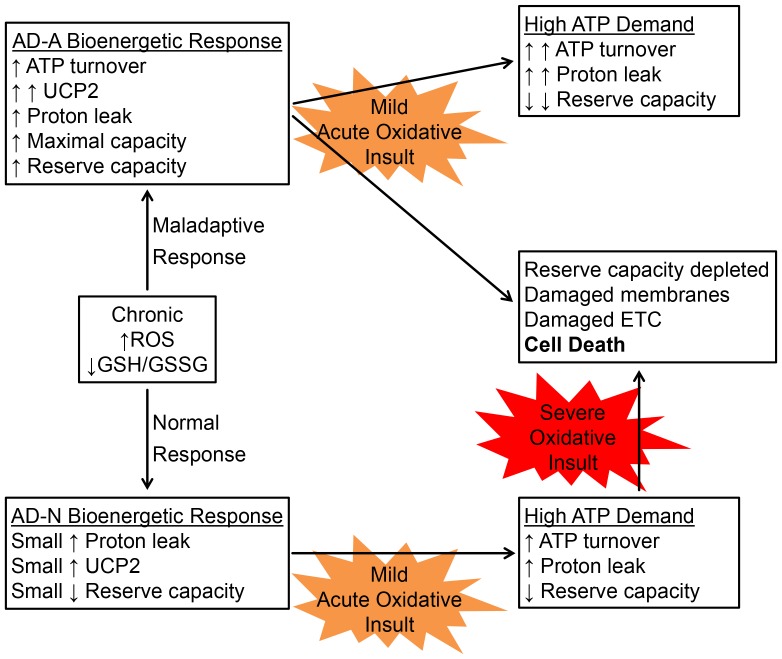
Normal adaptive and maladaptive mitochondrial responses to a more oxidized intracellular microenvironment. Diagrammed are the normal adaptive (AD-N) and maladaptive (AD-A) responses to a more highly oxidized intracellular microenvironment in the AD LCLs. The normal adaptive response of mitochondrial respiration to this more oxidized state, as seen in the AD-N LCLs, is to slightly decrease ATP turnover (ATP-linked respiration) and slightly increase proton leak, likely through a small increase in UCP2 expression (although not confirmed) resulting in a slight decrease in reserve capacity. In contrast, as seen in the AD-A LCLs, a maladaptive response is to significantly increase proton leak (through increased UCP2 expression) as well as ATP turnover, maximal respiratory capacity and reserve capacity. When then exposed to a mild oxidative insult, both groups experience higher ATP demand and respond by increasing ATP turnover and proton leak respiration, thereby reducing reserve capacity; however, this response is greatly exaggerated in the maladaptive AD-A LCLs. We propose that while all cells are vulnerable to an ATP crisis and cell death under severe oxidative stress conditions, that in the maladaptive AD-A LCLs only a mild insult would be required to push the cells to a state of ATP crisis.

We can evaluate the normal adaptive response in mitochondrial respiration to a chronic oxidized intracellular microenvironment by examining the mitochondrial parameters of the AD-N LCLs. At baseline, AD-N LCLs demonstrate a slightly decreased ATP-linked respiration and slightly increased proton leak respiration and a concomitant slight decrease in reserve capacity compared to control LCLs. Unlike the AD-A LCLs, the AD-N LCLs do not exhibit increased ATP-linked respiration and maximal respiratory capacity at baseline. Thus, we can consider the increased ATP-linked respiration and maximal respiratory capacity in the AD-A LCLs at baseline as maladaptive responses to a chronic oxidized intracellular microenvironment. These normal adaptive and maladaptive responses of the AD-N and AD-A LCL subgroups respectively and the expected outcomes when exposed to acute oxidative insults are diagramed in Figure 11. When exposed to mild acute oxidative insults, the two subgroups respond by increasing ATP turnover (ATP-linked respiration) and proton leak, thereby reducing the reserve capacity. However, this response is greatly exaggerated in the maladaptive AD-A LCLs, and occurs at a lower level of acute oxidative stress than in the AD-N LCLs. Thus, AD-A LCLs would be expected to respond to a secondary mild acute insult by going into ATP crisis leading to cell death, whereas we would expect that a more severe acute ROS insult would be required to push the AD-N LCLs to this point.

### Molecular Mechanisms Associated with the Increase in ATP-linked Respiration

Elevations in ATP-linked respiration in the AD-A LCL subgroup is consistent with clinical reports of electron transport chain (ETC) over-activity in ASD children. Frye and Naviaux [50] reported five ASD/MD children with complex IV over-activity and Graf et al [51] reported a ASD/MD child with complex I over-activity. The fact that ATP-linked respiration is increased at baseline suggests that increased ATP production may be an important cytoprotective mechanism against ROS in AD-A LCLs. Maximal respiratory capacity is a measure of the maximum ability of the ETC to generate ATP. Higher maximal respiratory capacity in the AD-A subgroup is consistent with an overall increase in ATP-linked respiration and, again, suggests an increased ATP demand and a compensatory over-activity of the ETC in the AD-A LCLs.

### Molecular Mechanisms Associated with the Increased Proton Leak Respiration

Proton leak reduces the mitochondrial membrane potential (MMP) which, in turn, decreases ETC ROS generation [52]. Proton leak is modulated by several mechanisms, including the adenine translocator and, in lymphocytes, UCP2 [30]. Given that UCP2 is up-regulated by chronic oxidative stress [31], [32] and that AD-A LCLs have chronic elevations in ROS, we examined whether UCP2 was up-regulated in the AD-A LCLs. We demonstrate for the first time that UCP2 content is indeed elevated in the AD-A LCLs, providing mechanistic insight into the abnormally elevated proton leak respiration and supporting chronic elevations in ROS in this subgroup.

To investigate the contribution of UCP2 to the abnormal mitochondrial respiratory function in the AD-A subgroup, we used genipin to inhibit UCP2. Genipin resulted in overall increases in all of the respiratory parameters, including proton leak respiration, and the increase in proton leak respiration with genipin was significantly higher in the AD-N subgroup. Since the cells were exposed to genipin for 24 hours prior to the assay, it is possible that other compensatory mechanisms were activated to up-regulate proton leak, possibly by promoting expression of adenine translocator isoforms involved in proton leak, or even running the ETC complexes backwards. Regardless, the AD-N LCLs were more capable of increasing proton leak when genipin was inhibited than the AD-A LCLs, suggesting a dependence on UCP2 in the AD-A LCLs or an inability to recruit any additional compensatory mechanisms to regulate ROS at the inner membrane perhaps because up-regulation of these mechanisms have already been exhausted. Alternatively, the increase in proton leak for the AD-N LCLs with genipin exposure could represent oxidative damage to the ETC complexes at the inner mitochondrial membrane. However, this latter possibility would be inconsistent with the fact that the AD-N subgroup was able to appropriately increase ATP production in response to an inhibition of UCP2 function.

There were important interactions between DMNQ and genipin whereby LCLs exposed to genipin exhibited a greater increase in ATP-linked and proton leak respiration and a greater decrease in maximal and reserve capacity with DMNQ than cells unexposed to genipin. This further suggests that the AD LCLs rely upon UCP2 to relieve excessive ROS production in the mitochondria. The most telling finding of the genipin experiments is that the AD-A subgroup, which exhibited higher baseline reserve capacity than AD-N subgroup, exhibited an even greater increase in reserve capacity with genipin compared to the AD-N group. Given that UCP2 content is higher in the AD-A subgroup, and inhibiting UCP2 with genipin further exacerbates the abnormal respiratory parameters, the AD-A LCLs appear to be particularly dependent on UCP2, likely to help counter excessive ROS production and maintain mitochondrial function. Relevant to the present findings, a recent study described an association with several mitochondrial genes and autism, including the gene coding for uncoupling protein 4 (UCP4), an isoform predominately expressed in the central nervous system [53].

An increase in MMP, potentially driven by ETC complex over-activity, could cause an increase in both ATP-linked and proton leak respiration and account for the findings in the AD-A subgroup [54]. However, we found no significant differences in MMP between the AD and control LCLs or the two AD subgroups. It is possible than an elevation in UCP2 content would have masked an increase in MMP in the AD-A subgroup; thus, further research is needed to clarify whether ETC complexes are overactive in this subgroup and whether such over-activity could drive an increase in ROS, MMP and UCP2 expression.

### Oxidative Stress in the AD LCLs

We measured the status of three separate redox couples as well as a marker of chronic oxidative stress (3-NT) and the amount of intracellular and mitochondrial ROS in the AD and control LCLs. Our previous findings of significantly decreased GSH and GSH/GSSG and increased intracellular ROS in AD LCLs relative to control LCLs were confirmed in this new age-matched and much larger sample size [22]. We also report for the first time that two additional redox couples are also significantly more oxidized in the AD LCLs relative to controls. The cysteine/cystine redox couple is typically considered the major extracellular redox buffer; however, reduced cysteine is the rate limiting amino acid required for GSH synthesis [55], so the shift towards more oxidized cystine in the AD LCLs further supports the more oxidized intracellular redox state of the AD LCLs relative to controls. We also found that the NADH/NAD^+^ ratio was lower (more oxidized) in the AD LCLs relative to controls. NADH, as an electron donor to the ETC is critically important for mitochondrial ETC function, and a shift towards more oxidized NAD^+^ may be a further indication of increased ETC activity in the AD LCLs.

While the three redox couples were significantly more oxidized in the AD LCLs compared to controls, they were not found to be significantly different between the two AD subgroups. However, intracellular ROS was significantly higher in the AD-A subgroup as compared to the AD-N subgroup. This confirms our previous finding that primary lymphocytes from children with AD exhibited higher intracellular ROS than lymphocytes from age-matched unaffected controls, and that the higher intracellular ROS in the primary lymphocytes from children with AD was driven by 5 out of 15 (33%) samples [24].

### Glycolytic Rates are Elevated in AD LCLs

Compared to controls, both basal ECAR and glycolytic reserve capacity were significantly elevated in the AD LCLs, but this elevation was particularly large for the AD-A LCL subgroup. The increased basal ECAR in the AD-A LCLs may be an attempt to increase anaerobic ATP production to meet higher ATP demands. Alternatively, the demand for glycolysis could simply be increased in the AD-A LCLs to provide pyruvate for ETC function, which appears to be higher in the AD-A LCLs. Nonetheless, the increase in both glycolysis and mitochondrial respiratory function in the AD LCLs, particularly the AD-A LCLs, is consistent with an increased demand for ATP likely due to chronically elevated oxidative stress in these cells. The glycolytic reserve capacity was overall very low for the LCLs indicating that they function at or near the maximal glycolytic capacity, which is not unexpected for transformed cell lines.

The dynamics of the change in glycolysis with DMNQ are tightly coupled to the change in the mitochondrial respiratory parameters. At the lowest concentration of DMNQ (5 µM), the LCLs respond by increasing mitochondrial oxygen consumption through both ATP-linked and proton leak respiration, and there is a simultaneous reduction in basal ECAR. Increased consumption of oxygen in the mitochondria requires ETC substrates; thus the decrease in ECAR is likely due to increased pull of pyruvate to acetyl-CoA by the mitochondria, resulting in a decreased conversion of pyruvate to lactate and an apparent reduction in ECAR. The glycolytic reserve at this DMNQ concentration increases due to the apparent reduction in the basal ECAR, as maximal ECAR does not change (data not shown). As the DMNQ concentration is further increased, the reserve capacity is depleted and ATP-linked respiration declines. Glycolytic reserve capacity also declines, which is due to a reduction in maximal ECAR with the higher DMNQ doses (data not shown). The simultaneous drop in ATP-linked respiration and glycolytic rates suggest that at the higher DMNQ concentrations, the ROS has significantly damaged bioenergetic components and the decreased the ability of the LCLs to make ATP by either mechanism.

### Mitochondrial Disease and Dysfunction in Autism Spectrum Disorder

The nature and prevalence of MD in ASD is still under investigation. A recent meta-analysis found that 5% of children with ASD meet criteria for a classic MD but that 30+% of children in the general ASD population exhibit biomarkers consistent with MD [8]. Recently, Frye demonstrated that 50+% of ASD children have biomarkers of MD that are consistently abnormal (i.e., repeatable) and valid (i.e., correlate with other MD biomarkers) [56]. In another study, 80% of the children with ASD demonstrated abnormal lymphocyte ETC function [57]. Interestingly some children with ASD/MD have ETC over-activity rather than ETC deficiencies [50], [51] and many ASD/MD cases do not manifest lactate elevation [50], [58], [59], a key biomarker commonly used to identify individuals with classic MD. This has raised the idea that children with ASD might have a type of mitochondrial dysfunction that is more prevalent and distinct from classic MD. This study has demonstrated a new type of mitochondrial dysfunction that may be the result of redox abnormalities and chronic oxidative stress and could affect a significant number of children with ASD. In fact, the LCL subgroup with mitochondrial abnormalities represented 32% of the total AD LCLs examined, a percentage similar to the prevalence of lactic acid elevation (a key marker of mitochondrial dysfunction) in ASD individuals as determined by a systematic meta-analysis [8]. It is well known that certain metabolic diseases are unmasked only during times of physiological stress. This study suggests that mitochondrial dysfunction in individuals with ASD may not manifest unless there are simultaneous ongoing physiological stressors. Thus, the results of clinical tests of mitochondrial disease in individuals with ASD may be very dependent on the physiological state of the individual at the time of testing, and collecting biomarkers of mitochondrial dysfunction may be most accurate during times of physiological stress such as fasting.

### Limitations

A limitation inherent in autism research is the availability of sufficient biological samples since there are no animal models that encompass the complete phenotype of autism; thus, we must utilize more readily available samples such as the LCLs employed in this study. Accumulating evidence indicates that autism includes broader systemic abnormalities including immune and redox abnormalities, oxidative stress and mitochondrial dysfunction [4]. Mitochondrial dysfunction, as an underlying defect, would affect high energy demanding systems, particularly the brain and immune system; thus, immune cells may be an ideal surrogate for investigating the consequences of mitochondrial abnormalities when neural tissue cannot be practically studied. It will be important to expand these findings to primary immune cells (PBMCs) with the aim of developing a model of mitochondrial function in an accessible tissue such as immune cells and developing a practical biomarker which could be eventually clinically useful. Finding mitochondrial dysfunction similar to that found in the LCLs in PBMC from a subgroup of individuals with ASD could validate the LCL model and further help to determine the clinical relevance of this type of mitochondrial dysfunction in individuals with ASD.

The number of subgroups that could be identified depended on the total number of LCLs examined, which was limited. Future studies will need to examine a larger number of LCLs to confirm these findings and determine whether there are multiple LCL subgroups. Furthermore, due to the limited numbers of LCLs available, we utilized only male-derived LCLs to reduce potential variability in the experiments and to increase the homogeneity of the samples; thus the potential effects of gender on mitochondrial function in the AD LCLs remains to be determined.

### Conclusions

This study has identified a novel pattern of oxidative stress-induced mitochondrial dysfunction in lymphoblastoid cells derived from AD children that appears to be present in a significant subgroup of LCLs. Thus, we demonstrate a new type of mitochondrial disorder that may affect a significant subgroup of AD children and provides insight into the interactions between systems that have been independently demonstrated to be abnormal in ASD [4]. This information provides insight into the pathophysiology associated with ASD and a pathway for designing medical treatments for ASD.
